# A Human Model of the Effects of an Instant Sheer Weight Loss on Cardiopulmonary Parameters during a Treadmill Run

**DOI:** 10.3390/jcm12010098

**Published:** 2022-12-22

**Authors:** Michał J. Pytka, Remigiusz A. Domin, Jacek L. Tarchalski, Marta I. Lubarska, Mikołaj S. Żołyński, Jan Niziński, Jarosław Piskorski, Andrzej Wykrętowicz, Przemysław Guzik

**Affiliations:** 1 Department of Cardiology—Intensive Therapy, Poznan University of Medical Sciences, Ul. Przybyszewskiego 49, 60-355 Poznan, Poland; 2 Department of Endocrinology, Metabolism and Internal Medicine, Poznan University of Medical Sciences, Ul. Przybyszewskiego 49, 60-355 Poznan, Poland; 3 Institute of Physics, University of Zielona Góra, Ul. Szafrana 4a, 65-516 Zielona Góra, Poland

**Keywords:** cardiopulmonary exercise test, cardiorespiratory function, exercise obesity, sports weight vest, physical performance, weight reduction

## Abstract

Exercise tolerance is limited in obesity and improves after weight reduction; therefore, we mutually compared the relative changes in exercise capacity variables during cardiopulmonary exercise tests (CPET) in a 12 kg sheer weight reduction model. Twenty healthy male runners underwent two CPETs: CPET1 with the actual body weight, which determined the anaerobic threshold (AT) and respiratory compensation point (RCP); and CPET2 during which the participants wore a +12 kg vest and ran at the AT speed set during the CPET1. Running after body weight reduction shifted the CPET parameters from the high-mixed aerobic-anaerobic (RCP) to the aerobic zone (AT), but these relative changes were not mutually similar. The most beneficial changes were found for breathing mechanics parameters (range 12–28%), followed by cardiovascular function (6–7%), gas exchange (5–6%), and the smallest for the respiratory exchange ratio (5%) representing the energy metabolism during exercise. There was no correlation between the extent of the relative body weight change (median value ~15%) and the changes in CPET parameters. Weight reduction improves exercise capacity and tolerance. However, the observed relative changes are not related to the magnitude of the body change nor comparable between various parameters characterizing the pulmonary and cardiovascular systems and energy metabolism.

## 1. Introduction

Excess weight and obesity are risk factors for diabetes, hypertension, ischemic heart disease, stroke, sleep apnea, cancer, as well as other lifestyle diseases [1,2,3,4,5]. Excessive intake of calories and physical inactivity are the major causes of excess weight [6], so complex lifestyle changes (reduced calorie intake and increased physical activity) combined with bariatric surgery and/or pharmacological treatment can reduce body weight.

Lifestyle modification can decrease body weight by up to 10% [7], and a 15–16% decrease can be achieved by the combination of a low-calorie diet, exercise, and a glucagon-like peptide-1 analog [8,9,10,11], whereas invasive weight reduction, such as bariatric surgery, can further decrease body mass up to 22% [12,13].

Body weight reduction is a long-term process impossible to achieve without surgical intervention within a couple of days. As already mentioned, a combination of lifestyle change with a current pharmacological treatment results, on average, in a 15–16% weight reduction after a year [8,9,10,11]. Many adaptive changes go beyond decreasing fat, water, and lean tissue amount during weight reduction. Among these changes are metabolic and hormonal adaptations, including improved insulin sensitivity, reduced fasting and post-prandial glucose concentrations, circulating triglycerides, and LDL and HDL cholesterol levels [14,15].

Improved exercise tolerance is the most common beneficial effect of weight loss [16,17,18] and is determined by mutual interactions of the cardiovascular and respiratory systems, efficient tissue oxygen supply, and metabolite and heat removal from the working muscles. These effects can be measured during exercise by the cardiopulmonary exercise test (CPET) [19].

Obese individuals have higher oxygen consumption (VO_2_), breathing frequency (BF), and heart rate (HR) than lean individuals [20]. More than 5% body weight reduction can achieve measurable changes in these and other CPET parameters such as respiratory exchange ratio (RER), exhaled carbon dioxide volume (VCO_2_), or minute ventilation (VE) [21]. 

However, it is unclear whether weight reduction comparably affects various functional exercise tolerance contributors. In a clinical model of long-lasting weight reduction, many factors (see above), not directly related to the sheer mass effect, influence the CPET. We, however, were interested in the potential effect of sheer weight reduction on parameters describing exercise tolerance. 

It is impossible to develop a model of an instant sheer weight reduction for studying exercise tolerance. Therefore, we assumed that the target body weight after reduction should be the real and actual body weight, while the artificially increased by approximately 15% of the total mass body weight might reflect the pre-reduction stage. To achieve it, we developed a model of a simulated “instant” 12 kg sheer weight reduction in fit male runners. With this model, we studied the relative changes in CPET parameters. We compared a treadmill run with 12 kg weight vests with a treadmill run with the actual body weight on two separate days to simulate instant weight reduction.

## 2. Materials and Methods

### 2.1. Participants

Twenty healthy volunteer adult male amateur long-distance runners aged 18–50 years were recruited. All participants trained a minimum of three times a week, covering a total weekly distance of at least 30 km, and were experienced, long-distance runners. All participants provided written informed consent before study participation and were informed that they could withdraw from the study at any time. The study protocol was approved by the Bioethical Committee at the Poznan University of Medical Sciences (decision number 365/21 on 6 May 2021), and the project was conducted according to the Declaration of Helsinki [22]. All data were collected, stored, and analyzed with strict confidentiality in the Redcap data capture tools hosted at Poznan University of Medical Sciences (https://redcap.ump.edu.pl, accessed on 29 November 2022). All data were anonymized for storage and analysis. 

### 2.2. Clinical Evaluation and Echocardiography

A physician took the medical history and performed a physical examination, including body weight, height, and training details. All participants underwent transthoracic echocardiography (Vivid E95 or E9, General Electric Company, Boston, MA, USA) performed by experienced physicians as per the American Society of Echocardiography guidelines [23]. The images and cine loops were recorded from typical transthoracic views and used for the post-processing analysis and measurements with the TOMTEC Imaging Systems GmbH, Unterschleissheim, Germany (distributed by Phillips, Amsterdam, The Netherlands).

### 2.3. Resting Spirometry and Cardiopulmonary Exercise Testing

#### 2.3.1. General Settings and Resting Spirometry

**Figure 1:**
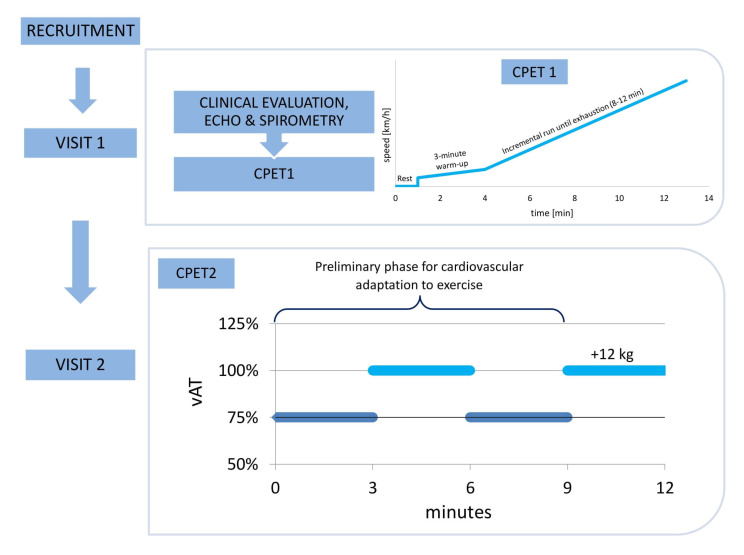
The study flow. Clinical evaluation, echocardiography (ECHO), and spirometry were conducted on VISIT 1, and the progressive cardiopulmonary exercise test (CPET1) was individually tailored to consist of rest, a 3 min warm-up, and an incremental run until exhaustion, aiming to last between 8 and 12 min. On VISIT 2, the second cardiopulmonary exercise test (CPET2) was performed with the exercise intensity set individually for each participant, with the treadmill velocity set at the speed corresponding to the anaerobic threshold (vAT) established during CPET1. For cardiovascular adaptation, each runner ran without the weight vest at 75% vAT for 3 min, then at 100% vAT for 3 min, and at 75% vAT for 3 min. After this 9 min adaptation to the exercise, each runner put on the 12 kg weight vest and ran for 3 min.

Participants underwent two CPETs on a treadmill (Trackmaster TMX428, FULLVISION INC., Newton, KS, USA) on two separate days (visit 1—CPET1 and visit 2—CPET2). All CPET parameters were measured breath—by—breath by Blue Cherry, Geraterm Respiratory GmbH, Bad Kissingen, Germany. Figure 1 presents the study flow.

Resting spirometry was performed before the CPET1 to measure the forced expired volume in one second (FEV1) and derive the maximal voluntary ventilation (MVV) using the formula FEV1 × 40, which is necessary to calculate the breathing reserve (BR) [24]. 

#### 2.3.2. CPET1

CPET1 was performed until exhaustion with a ramp protocol individually tailored to each participant’s anticipated exercise capacity [25,26]. After acquiring the resting state, all participants ran on a treadmill for 3 min to warm up. Next, the progressive exercise running phase started with an incremental speed of the treadmill. The protocols for the incremental CPET phase were personalized and based on each individual’s current fastest pace of a 1 km run. The corresponding speed was set as the 10 min target for the treadmill so that the incremental phases of most of the CPETs were within the range of 8 to 12 min. All participants were encouraged to run until maximal exhaustion, and the test was stopped at their will. During the post-exercise recovery, participants sat on a chair for 5 min. The treadmill angle was set at 1% elevation for the warm-up and incremental phases.

During CPET1, two ventilatory thresholds were determined [20,27]. The first ventilatory or anaerobic threshold (AT) was estimated by three methods in the following order, i.e., the V-slope from the VO_2_ vs. VCO_2_ relationship; VO_2_ equivalent (VE/VO_2_), and end-tidal partial O_2_ pressure (PetO_2_) vs. treadmill speed. The second ventilatory threshold or respiratory compensation point (RCP) was estimated with the PetCO_2_ vs. treadmill speed, VE/VCO_2_ V-slope from the VE vs. VCO_2_ plot, and VCO_2_ equivalent (VE/VCO_2_) vs. treadmill speed. AT and RCP thresholds were determined as the best agreement between at least two physicians analyzing the same CPET result. The CPET1 was summarized by measures taken at rest, AT, RCP, and the peak exercise.

#### 2.3.3. CPET2

CPET2 was performed on another day within a week following the CPET1. Participants re-took the CPET according to the following individualized protocol for each runner, and the treadmill speed recorded at AT (vAT) during CPET1 was set as the target speed of the CPET2. We calculated 75% of vAT, which was used as an adaptation phase speed in CPET2. During the whole CPET2, the treadmill angle was set at 1%. The preliminary phase, designed for cardiovascular adaptation to exercise, consisted of a 3 min run at 75% vAT, followed by a 3 min run at 100% vAT. Next, the volunteers ran for 3 min at 75% vAT (preparation phase), after which they stepped from the treadmill for up to 15 s to put the +12 kg vest on, then returned to the treadmill to run for another 3 min at 100% vAT. Figure 1 shows the study flow.

The following standard CPET parameters were measured breath by breath [28]:

- HR—heart rate.
- VO_2_—the volume of consumed O_2_.
- O_2_ pulse as a ratio of VO_2_ to HR.
- VCO_2_—the volume of produced CO_2_.
- VE—minute ventilation.
- TV—the tidal volume.
- BF—breathing frequency.
- BR%—breathing reserve as a fraction of VE to MVV.
- VE/VO_2_—the ventilatory equivalent for oxygen.
- VE/VCO_2_—the ventilatory equivalent for carbon dioxide.
- RER—respiratory exchange ratio.
- PetO_2_—the end-tidal oxygen tension.
- PetCO_2_—the end-tidal carbon dioxide tension.

For further analysis, we used the mean values of CPET variables measured within 15 s of AT and RCP (from CPET1), and the last 30 s of the 3 min run with the +12 kg vest (from CPET2). The CPET2 measures collected during the run at vAT with the +12 kg vest were compared with the CPET1 parameters at AT and RCP. Our primary intention, however, was to study the potential effects of simulated “instant” 12 kg weight reduction on CPET parameters, particularly those corresponding to exercise tolerance. Therefore, the relative changes between the runs with the +12 kg vest at vAT versus the unloaded CPET1 run at AT and RCP were compared using the CPET2 parameters measured during the +12 kg vest run as reference:

$$Parameter_{rc}=\frac{Parameter_{stage}-Parameter_{12\mathrm{kg}}}{Parameter_{12\mathrm{kg}}}\times 100\%$$

where *Parameter_rc_* is the relative change in a specific parameter compared to the run with the +12 kg vest; *Parameter_stage_* is the absolute value of a specific parameter recorded during CPET1 at various stages (AT, RCP); and *Parameter*_12kg_ is the absolute value of a specific parameter recorded during the CPET2 run with the +12 kg vest.

### 2.4. Statistical Analysis

Due to the non-Gaussian data distribution (by the D’Agostino-Pearson test), data were summarized as median and the 25th and 75th percentile. The post-hoc Dunn–Bonferroni test was used to compare absolute values and their relative changes between both tests. Non-parametric Spearman correlation with the rho coefficient was used to describe the association between the relative change in body weight with the relative changes in the CPET parameters between CPET2 and CPET1 tests. A *p*-value <0.05 was considered significant. Statistical analyses were performed using PQStat Software (PQStat v.1.8.4.124, PQStat, Poznań, Poland).

## 3. Results

### 3.1. Baseline Clinical Characteristics, Echocardiography, and Resting CPET 

Table 1 summarizes the clinical characteristics of all participants. Their median age was 26 years. All exercised regularly with a median of five training days per week with eight training hours per week. Echocardiography revealed median values of the diameters of the left ventricle, right ventricle, and right atrium, and the left ventricular wall thickness to be normal. The left ventricle end-systolic diameter was in the upper limit—40.5 mm, and the adaptive dilation of the left atrium was nearly 31 mL/m^2^. Descriptors of the left ventricular systolic and diastolic function were in the normal range, with no hemodynamically and clinically relevant valvular diseases.

Resting spirometry and CPET values were within the normal range, with a median breathing frequency of 17/min, breathing reserve of 92%, VCO_2_ of 0.38 L/min, and VO_2_ of 0.46 L/min. The calculated RER was 0.85, the resting HR was 79/min, and the O_2_ pulse was 5.8 mL/beat. The median peak treadmill speed during incremental exercise was over 17 km/h. The HR increased to 184 beats/min, BF to 51/min, TV to 2.7 L, and VE to 146 L/min. Additionally, RER reached 1.17, indicating adequate maximal effort of the volunteers. The median VO_2_ peak reached 4.54 L/min, and the peak VCO_2_ was 3.83 L/min. At peak exercise, the runners achieved an O_2_ pulse of 18.9 mL/beat. The CPET results at rest and peak exercise are shown in Table 2.

Our model demonstrates the potential effects of a simulated “instant” 12 kg weight reduction. Therefore, the CPET test results are presented in a reverse mode, first from CPET2 and then from CPET1 during AT and RCP. The median BMI of the runners with the +12 kg vests was 25.8 kg/m^2^, and after the simulated weight reduction, it decreased to 22.2 kg/m^2^. With the +12 kg vests, 4 runners became obese, 11 were overweight, and 5 had normal BMI. After the simulated weight reduction, 13 runners had normal BMI, 7 were overweight, and none were obese. The median value of simulated relative body weight reduction was ~15% for the whole group.

**Figure 2:**
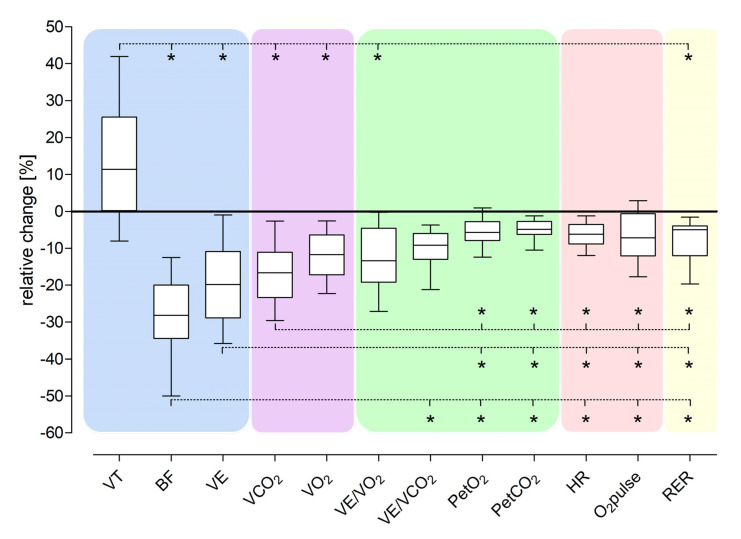
Comparison of relative changes of CPET parameters from CPET2 (run with vAT with +12 kg weight vest) and CPET1 (AT). * Statistically significant differences between relative changes of pairs of CPET parameters. AT—anaerobic threshold; BF—breathing frequency; CPET—cardiopulmonary exercise test; HR—heart rate; O_2_pulse—the ratio of VO_2_ to HR; PetCO_2_—the end-tidal carbon dioxide tension; PetO_2_—the end-tidal oxygen tension; RC—relative change; RER—respiratory exchange ratio; TV—the tidal volume; vAT—treadmill speed recorded at AT during CPET1; VCO_2_—the volume of produced CO_2_, VE—minute ventilation; VE/VCO_2_—the ventilatory equivalent for carbon dioxide; VE/VO_2_—the ventilatory equivalent for oxygen; VO_2_—the volume of consumed O_2_.

The absolute values of parameters from CPET2 for the +12 kg vest run and from CPET1 (AT and RCP) are presented in Table 3. The +12 kg run at the vAT during the CPET2 was the reference for comparisons with the run at the vAT during the CPET1 (Table 4). Mutual comparisons of all relative changes (Table 5) were made to investigate the proportionality of these changes (Figure 2).

**Table 3:** Absolute values of CPET parameters during CPET2 (run with vAT with +12 kg weight vest) and CPET1 (AT, RCP).

	CPET2			CPET1 AT			vs. AT	CPET1 RCP			vs. RCP
	Median	25th p	75th p	Median	25th p	75th p	*p*	Median	25th p	75th p	*p*
BF (breaths/min)	47.0	44.0	50.5	35.8	31.0	39.1	<0.0001	43.0	41.0	46.5	0.6681
VCO_2_ (L/min)	3.41	2.97	3.67	2.72	2.52	3.02	0.0423	3.84	3.41	4.17	0.3003
VE/VCO_2_	29.15	27.83	30.53	26.41	24.87	27.70	<0.0001	28.50	27.48	29.55	1
VE/VO_2_	30.05	28.30	32.55	27.15	24.78	28.38	0.0023	30.95	29.33	33.13	1
VO_2_ (L/min)	3.27	2.86	3.48	2.81	2.58	3.11	0.0132	3.50	3.15	3.80	1
VE (L/min)	104.40	92.83	117.84	80.52	73.94	87.30	0.0197	114.02	104.78	128.45	0.6681
TV (L)	2.24	1.98	2.38	2.43	2.24	2.62	0.3972	2.60	2.45	2.77	0.0005
RER	1.04	1.01	1.09	0.96	0.93	0.99	0.0132	1.09	1.05	1.13	0.8499
HR (beats/min)	169	167	178	163	156	166	0.0291	178	174	185	0.3459
O_2_pulse (mL/beat)	18.86	16.91	20.50	17.58	16.27	19.06	0.0132	19.59	17.57	22.64	1
PetO_2_ (mmHg)	111.0	108.0	112.3	107.0	100.4	108.0	0.0107	111.5	109.8	113.3	1
PetCO_2_ (mmHg)	41.0	40.4	43.0	39.1	38.2	40.0	0.0001	40.0	38.8	42.0	0.224

AT—anaerobic threshold; BF—breathing frequency; CPET—cardiopulmonary exercise test; HR—heart rate; O_2_pulse—the ratio of VO_2_ to HR; PetCO_2_—the end-tidal carbon dioxide tension; PetO_2_—the end-tidal oxygen tension; RCP—respiratory compensation point; RER—respiratory exchange ratio; TV—the tidal volume; vAT—treadmill speed recorded at AT during CPET1; VCO_2_—the volume of produced CO_2_, VE—minute ventilation; VE/VCO_2_—the ventilatory equivalent for carbon dioxide; VE/VO_2_—the ventilatory equivalent for oxygen; VO_2_—the volume of consumed O_2_.

**Table 4:** Relative changes between CPET parameters from CPET2 (run with vAT with +12 kg weight vest) and CPET1 (AT, RCP).

**%**	AT			RCP		
	Median	25th p	75th p	Median	25th p	75th p
BF	−28.13	−32.94	−20.00	−4.36	−12.15	0.00
VCO_2_	−16.64	−23.21	−12.55	12.06	5.52	20.98
VE/VCO_2_	−9.18	−11.99	−6.03	−3.09	−6.83	0.35
VE/VO_2_	−13.34	−17.89	−6.71	4.02	−2.95	7.97
VO_2_	−11.69	−17.16	−8.23	8.40	0.72	15.35
VE	−19.84	−28.50	−13.16	11.18	2.40	19.17
TV	11.35	2.82	20.67	21.55	4.89	27.65
RER	−4.96	−11.80	−4.23	4.62	1.22	9.56
HR	−6.21	−8.44	−3.57	4.73	2.89	6.84
O_2_ pulse	−7.18	−11.94	−1.97	1.59	−2.83	10.09
PetO_2_	−5.69	−7.66	−2.82	0.92	−0.90	2.08
PetCO_2_	−4.88	−5.53	−2.88	−3.36	−4.88	0.00

AT—anaerobic threshold; BF—breathing frequency; CPET—cardiopulmonary exercise test; HR—heart rate; O_2_ pulse—the ratio of VO_2_ to HR; PetCO_2_—the end-tidal carbon dioxide tension; PetO_2_—the end-tidal oxygen tension; RCP—respiratory compensation point; RER—respiratory exchange ratio; TV—the tidal volume; vAT—treadmill speed recorded at AT during CPET1; VCO_2_—the volume of produced CO_2_, VE—minute ventilation; VE/VCO_2_—the ventilatory equivalent for carbon dioxide; VE/VO_2_—the ventilatory equivalent for oxygen; VO_2_—the volume of consumed O_2_.

**Table 5:** Comparison of relative changes of CPET parameters from CPET2 (run with vAT with +12 kg weight vest) and CPET1 (AT).

*p*-Value	BF	VCO_2_	VE/VCO_2_	VE/VO_2_	VO_2_	VE	TV	RER	HR	O_2_pulse	PetO_2_
VCO_2_	1	-	-	-	-	-	-	-	-	-	-
VE/VCO_2_	0.0347	1	-	-	-	-	-	-	-	-	-
VE/VO_2_	0.2909	1	1	-	-	-	-	-	-	-	-
VO_2_	0.6629	1	1	1	-	-	-	-	-	-	-
VE	1	1	0.7417	1	1	-	-	-	-	-	-
TV	<0.0001	<0.0001	0.0004	<0.0001	<0.0001	<0.0001	-	-	-	-	-
RER	0.0003	0.0917	1	1	1	0.0166	0.0400	-	-	-	-
HR	0.0001	0.0300	1	1	1	0.0048	0.1197	1	-	-	-
O_2_pulse	<0.0001	0.0224	1	1	1	0.0035	0.1553	1	1	-	-
PetO_2_	<0.0001	0.0011	1	0.4695	0.2004	0.0001	1	1	1	1	-
PetCO_2_	<0.0001	0.0013	1	0.5274	0.2272	0.0001	1	1	1	1	1

AT—anaerobic threshold; BF—breathing frequency; CPET—cardiopulmonary exercise test; HR—heart rate; O_2_pulse—the ratio of VO_2_ to HR; PetCO_2_—the end-tidal carbon dioxide tension; PetO_2_—the end-tidal oxygen tension; RCP—respiratory compensation point; RER—respiratory exchange ratio; TV—the tidal volume; vAT—treadmill speed recorded at AT during CPET1; VCO_2_—the volume of produced CO_2_, VE—minute ventilation; VE/VCO_2_—the ventilatory equivalent for carbon dioxide; VE/VO_2_—the ventilatory equivalent for oxygen; VO_2_—the volume of consumed O_2_.

### 3.2. Comparison with the CPET1 Run at AT

The simulated 12 kg weight reduction was associated with decreases in the absolute values of BF, VE, HR, RER, VCO_2_, VO_2_, O_2_ pulse, VE/VCO_2_, VE/VO_2_, PetCO_2,_ and PetO_2_ No change was observed in the TV (Table 3). Specifically, BF decreased from 47 breaths/min to 36 breaths/min, VE decreased from 104 L/min to 81 L/min, and TV increased from 2.2 L to 2.4 L (Table 3). 

### 3.3. Comparison with the CPET1 Run at RCP

Compared to the RCP, the simulated 12 kg weight reduction caused a decrease only in TV. No other parameters differed significantly between CPET2 and CPET1 at RCP (Table 3).

### 3.4. Relative Changes of CPET Parameters between CPET2 and CPET1 at AT

The relative changes of different CPET parameters caused by the simulated 12 kg weight reduction were not comparable between CPET2 and CPET1 at AT (Table 4 and Table 5). CPET parameters describing respiratory function changed more than the remaining variables. The median BF decline was 28%, VCO_2_ 17%, and VO_2_ 12%. There were modest improvements in the cardiovascular parameters, i.e., HR declined by 6% and O_2_ pulse by 7%. The smallest alterations for the relative CPET changes were found for RER (a decrease of 5%), and the PetO_2_ and PetCO_2_ (values decreased by nearly 6% and 5%, respectively). 

### 3.5. Comparison of Relative Changes of CPET Parameters between CPET2 and CPET1 at AT

Table 4 and Table 5 and Figure 2 show that the relative changes in CPET parameters between CPET2 and CPET1 at AT are not proportional, with the largest differences observed between BF and TV, O_2_ pulse, PetO_2_, PetCO_2_, TV and VE/VO_2_, VO_2_, VCO_2_, VE (all *p* < 0.0001). In Figure 2*,* all relative changes in CPET parameters are grouped into the following sets: ventilatory (blue area), mixed (ventilatory–cardiovascular) (violet area), gas exchange (green area), cardiovascular (red area), and metabolic (yellow area). Relative changes in ventilatory parameters significantly differed from the changes in other parameters. 

### 3.6. Correlation of Relative Changes of CPET Parameters (between CPET2 and CPET1 at AT) and Change in Weight

Table 6 shows no statistically significant correlations of relative CPET parameter changes (between CPET2 and CPET1 at AT) with the simulated body weight reduction percentage. CPET parameter changes between CPET2 (run with +12 kg vest) and CPET1 (during AT) are not in the same proportion and are independent of body weight reduction. 

## 4. Discussion

Our model shows that a simulated 12 kg weight reduction significantly changed most CPET variables in male runners, with decreases in BF, VE, HR, RER, VCO_2_, VO_2_, O_2_ pulse, VE/VCO_2_, VE/VO_2_, PetO_2_, and PetCO_2_, and insignificant increase in VT, when compared to the run at the same speed of vAT, but the relative changes were not of comparable proportion. There was no association between the magnitude of CPET parameters change and the body weight reduction percentage. CPET parameters for running at AT with +12 kg body weight were comparable to a faster run at RCP but without additional weight load, suggesting that ~15% body weight reduction in male runners shifts the respiratory, cardiovascular, and metabolic responses from the high mixed (aerobic–anaerobic) zone associated with metabolic acidemia close to RCP to a typical aerobic zone. 

### 4.1. Breathing Mechanics

The simulated ~15% weight reduction affects most CPET parameters, but the observed changes were not comparable among various CPET parameters describing exercise tolerance. Breathing frequency declined by nearly 30%, VE, VCO_2_ VE/VO_2,_ VO_2_ over 10%, and VE/VCO_2_ by nearly 10%, whereas the tidal volume increased by 11%. Balmain et al., suggested altered respiratory mechanics responsible for increased VE and PetCO_2_ and decreased VE/VCO_2_ slope during exercise in obese people [29]. During incremental exercise load, normal-weight runners reduce their dead space, whereas obese individuals have a higher physiological dead space and dead-space-to-ventilation ratio than normal-weight individuals [30]. The dead space is increased, and its impact on total lung capacity compared to ventilation is also increased; therefore, in obese runners, the dead space may not be reduceable, and their respiratory muscles must perform more work to remove the same amount of CO_2_ and compensate for metabolic acidemia [29,31]. Additionally, obese individuals produce even more CO_2_ during physical effort than nonobese people. Borasio et al., showed that after weight reduction due to sleeve gastrectomy, the lung volumes and flows during forced expiration at rest improve, and resting ventilation and tidal volume are reduced. During exercise, there is decreased ventilation and a shallower ventilatory pattern (lower BF and larger TV response). Additionally, after weight loss, dead space ventilation and saturation increase [32]. 

Poorer chest and abdominal wall compliance and inertia also contribute to impaired respiratory mechanics typical for obesity. Richman et al. [33] showed that obese subjects with unilateral diaphragm paralysis had more reduced peak VO_2_/kg and VE than subjects with either obesity or unilateral diaphragm paralysis. Obese people also have shallower breathing (TV), which needs to be compensated by increased BF [31,32].

Sport weight vests mimic the effects of obesity and fat tissue on breathing mechanics in several ways. First, the vests limit the expansion of the chest, abdominal walls, and shoulder movements. Second, the extra weight creates additional force vectors that need to be compensated by the chest and abdominal wall muscles. Third, the force vectors directed towards the abdomen resemble the effects of the abdominal wall and visceral fat, which reduce the extent of the diaphragm excursion up and down. Therefore, the thorax volume for expanding lungs during breathing becomes limited. All these changes correspond to the effects of lung restriction. In obesity, the ventilatory work is increased due to increased abdominal pressure and added mass on the chest wall, limiting the diaphragmatic movements during respiration and reducing the inspiratory capacity, tidal volume, and resting end-expiratory lung volume [20]. 

In our study, several indices of breathing mechanics improved during exercise after the simulated model of body weight reduction. Compared with the extra +12 kg weight, after taking off the sports vest, runners reduced their BF, VE, and VE/VCO_2_ but increased TV. Improving end-inspiratory lung volume by reducing dead space is one of the primary mechanisms of increasing TV during exercise in healthy and nonobese people. The effectiveness of such mechanisms is compromised both in obese persons and while running with the +12 kg vests [31].

Bhammar et al., found decreased BF, VE, VO_2_, and VCO_2_ during light exercise (cycling with the 60 W load) after an average 8% body weight reduction (15% in total fat mass) in a group of obese women undergoing a 12-week restrictive diet intervention [34]. Additionally, their resting total lung capacity (TLC), functional residual capacity (%TLC), and expiratory reserve volume (%TLC) increased, while inspiratory capacity (%TLC) decreased. After the weight reduction, TV during the 60W exercise decreased from 1.66 L to 1.57 L, possibly caused by the 60 W exercise being low-intensity exercise which does not challenge the respiratory system so much to substantially increase ventilation. Therefore, after the weight reduction, the TV was lower, but at peak exercise, after weight reduction, the TV increased from 1.9 L to 2.0 L [34]. Our model of simulated weight reduction also showed a decline in BF, VE (less respiratory work), and VE/VCO_2_ (smaller dead space), and improved TV (less lung restriction); therefore, losing body weight seems to have the most beneficial effects on breathing mechanics.

Obese people have a shallow and rapid breathing pattern due to increased work of the inspiratory muscles and reduced lung and chest wall compliance [31]. This forces these muscles to work harder, which requires higher O_2_ consumption and energy requirements. In healthy subjects, the oxygen cost of breathing (percentage of O_2_ consumed by the respiratory muscles) is below 5%. [35]. Losing weight decreases breathing costs (from 8.5% to 6.7% of total body VO_2_ after 8% body mass or 15% fat mass reduction) [34], thereby contributing to improved respiratory system functioning. Naturally, increased body weight also impacts leg muscles which have to carry out most of the physical work and carry a heavier person. A higher metabolic cost of moving a heavier body and legs and compensating for impaired breathing mechanics increases VO_2_ [33]. 

### 4.2. O_2_ Consumption and CO_2_ Production

Balmain et al., reported that obese people have higher metabolic and oxygen demands during exercise [29]. Obese adults also have elevated respiratory rates, smaller tidal volumes, and slightly elevated ventilatory equivalent for oxygen [30], with greater VO_2_ than normal-weight individuals during rest and exercise, possibly due to the additional metabolic demand required to move additional mass [30,33]. When VO_2_ is normalized to body mass, obese people have decreased VO_2_/kg [20], which could hypothetically be interpreted as increased metabolic demand of tissues when a runner has a lower body mass or higher efficiency of O_2_ utilization. However, obese people have similar or even slightly higher peak oxygen consumption during CPET than lean individuals [36,37]. Therefore, the change in VO_2_/kg must result from higher body mass. Submaximal exercise in obese subjects demands a larger peak VO_2_, indicating increased oxygen cost to exercise with a larger body mass [37]. In our “instant” weight loss model, we observed an increase in VO_2_/kg from 37.1 to 38.1 mL/kg/min (4% increase). However, these observations are complicated because this measurement is the effect of mathematical normalization of increased body mass. For this reason, we did not include these observations in our results. In our model, the body mass decreased when the volunteers took off the vests—the weight vests present a decrease in non-muscular, non-metabolic tissue—excess fat tissue and water, which does not take part in movement of the body. For more precise interpretation VO_2_ should be normalized to active metabolic tissue—muscle mass responsible for O_2_ consumption.

Obese individuals have increased muscle and fat mass, so additional energy is needed to move a larger body mass during exercise, and increased cardiorespiratory response is required to perform the same amount of energy [20]. This is in line with our findings, where an additional ~15% of body mass caused the metabolism to shift from AT to RCP with the same exercise load (treadmill speed). The other tissues, including muscles, which work harder, require a larger oxygen supply, which is provided by increased resting cardiac output per kilogram. Therefore, the cardiac output reserve for exercise is reduced [20]. Increased energy demand leads to increased CO_2_ production during exercise, and the VCO_2_ rose from 2.72 L/min to 3.41 L/min revealing the excess metabolic work. 

### 4.3. Energy Metabolism in Aerobic and Aerobic-Anaerobic Zones

Larger body mass requires more energy for working muscles, and anaerobic metabolism appears earlier when the more intense effort starts. Thus, in obesity, local muscle metabolic acidosis develops earlier [20]. Our model of the simulated “instant” 12 kg body weight reduction reversed this process and restored muscle metabolism to the aerobic zone by significantly reducing VCO_2_ by 21% and VO_2_ by 15%, whereas VCO_2_ reflects muscle CO_2_ production and VO_2_ measures muscle O_2_ consumption. In addition to lowering the body’s ventilatory demands discussed above, HR declined by over 6% and O_2_ pulse by 7%. Less body weight does not need to exploit the heart’s chronotropic (HR) and inotropic (O_2_ pulse as a surrogate of stroke volume) responses to exercise [18,26,36,37]. Oxygen ventilatory gas exchange is optimal when running at speed close to vAT. A decrease in O_2_pulse (i.e., VO_2_/HR) after the weight reduction suggests that the lungs absorb less oxygen with each heartbeat, also demonstrating that weight-reduction-induced changes in VO_2_ and HR are non-linear, i.e., one is declining faster than another.

After the weight reduction, RER, an energy source metabolism index, decreased by nearly 5% to <1. A lower RER means that the exercise is less intense, and more fat than glycogen is utilized in the aerobic oxygenation in mitochondria. It is a known but interesting phenomenon that more fat is burnt at the effort of lower than higher intensity when working muscle O_2_ demands are better covered [38]. If obese people intend to reduce fat, less intensive effort below the AT should be practiced, and for runners, this usually translates to slower runs. 

After weight loss, less energy is needed to perform an effort of similar intensity, such as running at the same speed. Other studies [38,39,40,41] and our results indirectly suggest that the duration of the effort, e.g., running, should be longer or at a higher intensity (speed) to burn the same amount of calories—while losing weight, the training plan should be regularly modified to continue fat and not glycogen mass reduction. 

### 4.4. Effects of Body Weight Reduction on PetO_2_

PetO_2_ during a run with a +12 kg vest at vAT was comparable to the faster run at vRCP without the extra weight. After the simulated weight reduction, PetO_2_ decreased by almost 6%. Physiologically, during exercise, the nadir PetO_2_ value is near AT when more efficient aerobic energy production predominates, and PetO_2_ increases with more intense effort above the AT [42].

The PetO_2_ nadir has two leading causes. The first is the already mentioned physiological ventilation improvement, whereas the second is the optimization of tissue oxygen extraction at this time. All O_2_ delivered to muscles is used by mitochondria to produce energy particles, e.g., ATP, from the oxidation of different molecules, including fatty acids and lactate. After this point, however, O_2_ delivery does not meet its tissue requirements, so cytoplasmic glycolysis gradually predominates over mitochondria in ATP production, lactates and H^+^ start accumulating, and local acidemia develops. The simulated weight reduction decreases PetO_2_ from near RCP to AT level, restores aerobic metabolism, and improves tissue O_2_ extraction, lactates and H^+^ are better balanced, and no metabolic acidemia occurs.

### 4.5. Effects of Body Weight Reduction on CPET Parameters

Nedeljkovic-Arsenovic et al., compared CPET parameters in obese patients before and six months after bariatric surgery [43], showing that only patients with body weight reduction over 18% had a decreased peak VO_2_ and improved ventilatory efficiency (described by increased BR, FEV1, and decreased peak VO_2,_ PetCO_2_). In our model, the approximate simulated 15% weight reduction was associated with improving many CPET parameters. Various clinical characteristics of studied people might explain the differences. For example, Nedeljkovic-Arsenovic et al., studied morbidly obese men and women (35% were physically active, but no definition of this term was provided in this study) with low exercise capacity, i.e., peak VO_2_ < 2.6 L/min, O_2_ pulse < 18 mL/beat, VE < 70 L/min, and RER no more than 1.07. Our study focused only on highly trained young male runners exercising regularly with above age- and gender-matched median exercise capacity indices, i.e., peak VO_2_ 3.8 L/min, O_2_ pulse 20.26 mL/beat VE 146 L/min [44,45]. However, our findings are similar to the observations of Wilms et al., who performed a CPET on a bicycle before and one year after bariatric surgery in obese adults [46]. Compared to presurgery, one year after this intervention, patients lowered their body weight by 27%, their HR during running at vAT was significantly lowered by 7.7%, and the peak VO_2_ and workload normalized to the body weight increased by 35% and 46%, respectively [46]. 

### 4.6. Limitations

For the studied weight reduction simulation, we used several approximations. First, we investigated the effects of sheer mass loss. However, the artificially increased body weight did not reflect natural fat, lean tissue, or water, typical components of excessive tissue in obese patients which are lost in various proportions during weight reduction. Second, weight reduction is prolonged with accompanying physiological changes and adaptations, but our “instant” weight loss model did not allow us to observe such adaptation. However, our model allowed us to investigate an isolated effect of body weight reduction on the CPET parameters. In this way, we excluded the potential impact of other metabolic and hormonal adaptations typically accompanying long-lasting weight reduction. Next, the +12 kg body weight was used as a reference so that the actual unloaded body weight might serve as a weight reduction result. Using such a model, we could study healthy runners with above-average exercise capacity (median values of peak VO_2_/kg 50.16 mL/kg, HR 184 beats/min, O_2_pulse 20.26 mL/beat, and RER 1.17) [44,45], who managed to run with extra +12 kg vests at the vAT pace for three minutes; people with poorer exercise capacity might be unable to complete such a task. Furthermore, extra-weight vests helped to mimic several effects of excessive fat on the chest and abdominal walls and breathing mechanics. Finally, we studied only 20 young male long-distance runners of the Caucasian race. Therefore, our findings cannot be extrapolated to both sexes, older people, those who do not train for long-distance running, and other ethnicities. 

## 5. Conclusions

After ~15% body weight reduction, running at the vAT speed shifts most CPET parameters from the near RCP to the AT threshold, but the magnitude of relative changes is neither proportional among various CPET parameters nor related to the weight loss percentage. The most beneficial alterations are observed in the breathing mechanics, followed by gas-exchange and cardiac parameters. The smallest relative changes are in RER, suggesting that running at the vAT speed utilizes more fat as an energy source when body weight is reduced. However, more research should be conducted with larger groups of various subjects. Body mass reduction techniques (including pharmacological treatment) should be constantly developed and widely propagated due to their many beneficial effects. 

## Figures and Tables

**Table 1:** Summary baseline characteristics of the healthy male amateur runners.

	Median	LQ	UQ
Age (years)	26	23	42
Training days a week (days)	5	4	7
Training hours a week (hours)	8	5	16
Height (cm)	182	175	184
Body mass (kg)	73.5	67.0	78.5
Delta mass (%)	14.04	13.26	15.19
BMI (kg/m^2^)	22.20	21.18	25.86
BMI +12 kg (kg/m^2^)	25.81	25.03	29.82
Treadmill velocity at AT (km/h)	12.4	11.7	13.0
Treadmill velocity at RCP (km/h)	15.50	14.48	16.15
Maximal treadmill velocity (km/h)	17.15	16.25	17.93
RVIDd (mm)	34.0	31.0	37.5
IVSd (mm)	9	8	9
LVIDd (mm)	50.0	47.5	54.5
LVPWd (mm)	9.0	9.0	10.5
LVIDs (mm)	40.5	34.0	42.0
LVEF (%)	63.2	61.4	64.4
LADs (mm)	38	34	41
MV E (m/s)	0.75	0.65	0.84
MV A (m/s)	0.44	0.41	0.53
E/E’ mean	4.83	4.70	5.47
AV Vmax (m/s)	1.19	1.10	1.31
LAI (mL/m^2^)	41.99	30.79	49.62

‘A’—velocity of the ‘a’ wave in pulse wave Doppler; AT—anaerobic threshold; AV Vmax—the maximal velocity of blood ejected through the aortic valve; BMI—body mass index; ‘E’—velocity of ‘e’ wave measured in tissue Doppler; IVSd—intraventricular septum diameter during diastole; LADs—left atrium diameter during systole; LAI—left atrial diameter during systole indexed to body surface area; LQ—lower quartile; LVEF—left ventricle ejection fraction; LVIDd—left ventricular internal diameter during diastole; LVIDs—left ventricular internal diameter during systole; LVPWd—left ventricular posterior wall diameter during diastole; MV—mitral valve; RCP—respiratory compensation point; RVIDd—right ventricle internal diameter during diastole; UQ—upper quartile.

**Table 2:** Summary baseline characteristics of CPET parameters at rest and peak exercise.

	Rest			Peak Exercise		
	Median	LQ	UQ	Median	LQ	UQ
BF (breaths/min)	17.0	13.3	21.0	50.5	47.5	59.5
VCO_2_ (L/min)	0.38	0.29	0.48	4.54	3.98	4.78
VE/VCO_2_	32.4	29.9	34.0	30.9	29.1	32.0
VE/VO_2_	27.0	23.9	29.6	36.8	32.9	38.2
VO_2_ (L/min)	0.46	0.37	0.54	3.83	3.46	4.12
VO_2_kg (L/min × kg)	5.92	5.23	7.21	50.16	47.49	52.69
VE (L/min)	14.33	9.97	16.42	146.41	126.48	160.39
TV (L)	0.84	0.68	1.15	2.72	2.51	3.01
RER	0.85	0.78	0.92	1.17	1.12	1.21
HR (beats/min)	79	70	88	184	181	195
O_2_pulse (mL/beat)	5.78	5.04	6.64	20.26	18.18	22.79
PetO_2_ (mmHg)	110.0	104.0	111.3	116.5	112.8	118.0
PetCO_2_ (mmHg)	35.5	34.5	37.0	37.5	36.8	40.0

BF—breathing frequency; CPET—cardiopulmonary exercise test; HR—heart rate; O_2_pulse—the ratio of VO_2_ to HR; LQ—lower quartile; PetCO_2_—the end-tidal carbon dioxide tension; PetO_2_—the end-tidal oxygen tension; RER—respiratory exchange ratio; TV—the tidal volume; UQ—upper quartile; VCO_2_—the volume of produced CO_2_, VE—minute ventilation; VE/VCO_2_—the ventilatory equivalent for carbon dioxide; VE/VO_2_—the ventilatory equivalent for oxygen; VO_2_—the volume of consumed O_2_; VO_2_kg—the volume of consumed O_2_ per kilogram of body weight.

**Table 6:** Relationship of relative CPET parameter changes with the percentage simulated weight reduction.

Correlation between the Relative Body Weight Reduction and CPET Parameters between Running with a +12 kg Vest at vAT and				
	Without the Vest at vAT		Without the Vest at vRCP	
	rho	*p*-Value	rho	*p*-Value
BF	−0.07	0.7821	0.00	0.9849
VCO_2_	−0.18	0.4410	0.09	0.7113
VE/VCO_2_	−0.24	0.3052	−0.12	0.6212
VE/VO_2_	0.04	0.8696	0.01	0.9824
VO_2_	−0.24	0.3067	−0.14	0.5573
VE	−0.16	0.4884	−0.09	0.6995
VT	−0.13	0.5900	−0.06	0.7931
RER	0.03	0.9095	0.24	0.3146
HR	0.02	0.9245	0.39	0.0871
O_2_ pulse	−0.31	0.1834	−0.22	0.3575
PetO_2_	−0.10	0.6646	−0.31	0.1767
PetCO_2_	−0.02	0.9332	0.06	0.8109

AT—anaerobic threshold; BF—breathing frequency; CPET—cardiopulmonary exercise test; HR—heart rate; O_2_pulse—the ratio of VO_2_ to HR; PetCO_2_—the end-tidal carbon dioxide tension; PetO_2_—the end-tidal oxygen tension; RCP—respiratory compensation point; RER—respiratory exchange ratio; TV—the tidal volume; vAT—treadmill speed recorded at AT during CPET1; VCO_2_—the volume of produced CO_2_, VE—minute ventilation; VE/VCO_2_—the ventilatory equivalent for carbon dioxide; VE/VO_2_—the ventilatory equivalent for oxygen; VO_2_—the volume of consumed O_2_; vRCP—velocity at respiratory compensation point during CPET1.

## Data Availability

The datasets generated and/or analysed for this study are currently not publicly available due to further ongoing analyses by the authors. Selected data, however, are available from the corresponding author upon request.

## References

[1] Guzik A., Bushnell C. (2017). Stroke Epidemiology and Risk Factor Management. Contin Lifelong Learn. Neurol.

[2] Mandviwala T., Khalid U., Deswal A. (2016). Obesity and Cardiovascular Disease: A Risk Factor or a Risk Marker?. Curr. Atheroscler. Rep..

[3] De Pergola G., Silvestris F. (2013). Obesity as a Major Risk Factor for Cancer. J. Obes..

[4] Aras M., Tchang B.G., Pape J. (2021). Obesity and Diabetes. Nurs. Clin. N. Am..

[5] Kachur S., Lavie C.J., de Schutter A., Milani R.V., Ventura H.O. (2017). Obesity and cardiovascular diseases. Minerva Med.

[6] Panahi S., Tremblay A. (2018). Sedentariness and Health: Is Sedentary Behavior More Than Just Physical Inactivity?. Front. Public Health.

[7] Wadden T.A., Butryn M.L., Wilson C. (2007). Lifestyle Modification for the Management of Obesity. Gastroenterology.

[8] Newsome P.N., Buchholtz K., Cusi K., Linder M., Okanoue T., Ratziu V., Sanyal A.J., Sejling A.S., Harrison S.A. (2021). A Placebo-Controlled Trial of Subcutaneous Semaglutide in Nonalcoholic Steatohepatitis. N. Engl. J. Med..

[9] Ojeniran M., Dube B., Paige A., Ton J., Lindblad A.J. (2021). Semaglutide for weight loss. Can. Fam. Physician.

[10] Lundgren J.R., Janus C., Jensen S.B.K., Juhl C.R., Olsen L.M., Christensen R.M., Svane M.S., Bandholm T., Bojsen-Møller K.N., Blond M.B. (2021). Healthy Weight Loss Maintenance with Exercise, Liraglutide, or Both Combined. N. Engl. J. Med..

[11] Grunvald E., Shah R., Hernaez R., Chandar A.K., Pickett-Blakely O., Teigen L.M., Harindhanavudhi T., Sultan S., Singh S., Davitkov P. (2022). AGA Clinical Practice Guideline on Pharmacological Interventions for Adults With Obesity. Gastroenterology.

[12] Grover B.T., Morell M.C., Kothari S.N., Borgert A.J., Kallies K.J., Baker M.T. (2019). Defining Weight Loss After Bariatric Surgery: A Call for Standardization. Obes. Surg..

[13] O’Brien P.E., Hindle A., Brennan L., Skinner S., Burton P., Smith A., Crosthwaite G., Brown W. (2019). Long-Term Outcomes After Bariatric Surgery: A Systematic Review and Meta-analysis of Weight Loss at 10 or More Years for All Bariatric Procedures and a Single-Centre Review of 20-Year Outcomes After Adjustable Gastric Banding. Obes. Surg..

[14] Petersen K.F., Shulman G.I. (2006). Etiology of Insulin Resistance. Am. J. Med..

[15] Tchernof A., Després J.-P. (2013). Pathophysiology of Human Visceral Obesity: An Update. Physiol. Rev..

[16] de Leiva A. (2009). What are the benefits of moderate weight loss?. Exp. Clin. Endocrinol. Diabetes.

[17] Vidal J. (2002). Updated review on the benefits of weight loss. Int. J. Obes..

[18] Rueda-Clausen C.F., Ogunleye A.A., Sharma A.M. (2015). Health Benefits of Long-Term Weight-Loss Maintenance. Annu. Rev. Nutr..

[19] Glaab T., Taube C. (2022). Practical guide to cardiopulmonary exercise testing in adults. Respir. Res..

[20] Sietsema K.E., Sue D.Y., Stringer W.W., Ward S.A. (2021). Wasserman & Whipp’s Principles of Exercise Testing and Interpretation.

[21] Gruchała-Niedoszytko M., Niedoszytko P., Kaczkan M., Pieszko M., Gierat-Haponiuk K., Śliwińska A., Skotnicka M., Szalewska D., Małgorzewicz S. (2019). Cardiopulmonary Excercise Test and Bioimpedance as prediction tools used to predict the outcomes of obesity treatment. Pol. Arch. Intern. Med..

[22] Sawicka-Gutaj N., Gruszczyński D., Guzik P., Mostowska A., Walkowiak J. (2022). Publication ethics of human studies in the light of the Declaration of Helsinki–A mini-review. J. Med. Sci..

[23] Mitchell C., Rahko P.S., Blauwet L.A., Canaday B., Finstuen J.A., Foster M.C., Horton K., Ogunyankin K.O., Palma R.A., Velazquez E.J. (2019). Guidelines for Performing a Comprehensive Transthoracic Echocardiographic Examination in Adults: Recommendations from the American Society of Echocardiography. J. Am. Soc. Echocardiogr..

[24] Stephens P., McBride M.G., Paridon S.M., Anderson R.H., Baker E.J., Penny D.J., Redington A.N., Rigby M.L., Wernovsky G. (2010). CHAPTER 20-Cardiopulmonary Stress Testing. Paediatric Cardiology.

[25] Myers J., Froelicher V.F. (1993). Exercise Testing. Cardiol. Clin..

[26] Kozlov S., Caprnda M., Chernova O., Matveeva M., Alekseeva I., Gazdikova K., Gaspar L., Kruzliak P., Filipova S., Gabbasov Z. (2020). Peak Responses during Exercise Treadmill Testing using Individualized Ramp Protocol and Modified Bruce Protocol in Elderly Patients. Folia Med. (Plovdiv).

[27] Wasserman K., Whipp B.J., Koyl S.N., Beaver W.L. (1973). Anaerobic threshold and respiratory gas exchange during exercise. J. Appl. Physiol..

[28] Kinnear W.J.M., Blakey J. (2014). A Practical Guide to the Interpretation of Cardiopulmonary Exercise Tests.

[29] Balmain B.N., Halverson Q.M., Tomlinson A.R., Edwards T., Ganio M.S., Babb T.G. (2021). Obesity Blunts the Ventilatory Response to Exercise in Men and Women. Ann. Am. Thorac. Soc..

[30] McMurray R.G., Ondrak K.S. (2011). Effects of being overweight on ventilatory dynamics of youth at rest and during exercise. Eur. J. Appl. Physiol..

[31] Bernhardt V., Babb T.G. (2016). Exertional dyspnoea in obesity. Eur. Respir. Rev..

[32] Borasio N., Neunhaeuserer D., Gasperetti A., Favero C., Baioccato V., Bergamin M., Busetto L., Foletto M., Vettor R., Ermolao A. (2021). Ventilatory Response at Rest and During Maximal Exercise Testing in Patients with Severe Obesity Before and After Sleeve Gastrectomy. Obes. Surg..

[33] Richman P.S., Yeung P., Bilfinger T.V., Yang J., Stringer W.W. (2019). Exercise Capacity in Unilateral Diaphragm Paralysis: The Effect of Obesity. Pulm. Med..

[34] Bhammar D.M., Stickford J.L., Bernhardt V., Babb T.G. (2016). Effect of weight loss on operational lung volumes and oxygen cost of breathing in obese women. Int. J. Obes..

[35] Kanak R., Fahey P.J., Vanderwarf C. (1985). Oxygen Cost of Breathing. Chest.

[36] Hakala K., Mustajoki P., Aittomäki J., Sovijärvi A. (1996). Improved gas exchange during exercise after weight loss in morbid obesity. Clin. Physiol..

[37] Hulens M., Vansant G., Lysens R., Claessens A.L., Muls E. (2001). Exercise capacity in lean versus obese women. Scand. J. Med. Sci. Sports.

[38] Wolfe R.R., Richter E.A., Kiens B., Galbo H., Saltin B. (1998). Fat Metabolism in Exercise. Skeletal Muscle Metabolism in Exercise and Diabetes.

[39] Hargreaves M., Spriet L.L. (2020). Skeletal muscle energy metabolism during exercise. Nat. Metab..

[40] Harris M.B., Kuo C.-H. (2021). Scientific Challenges on Theory of Fat Burning by Exercise. Front. Physiol..

[41] Achten J., Jeukendrup A.E. (2004). Optimizing fat oxidation through exercise and diet. Nutrition.

[42] Binder R.K., Wonisch M., Corra U., Cohen-Solal A., Vanhees L., Saner H., Schmid J.P. (2008). Methodological approach to the first and second lactate threshold in incremental cardiopulmonary exercise testing. Eur. J. Cardiovasc. Prev. Rehabil..

[43] Nedeljkovic-Arsenovic O., Banovic M., Radenkovic D., Rancic N., Polovina S., Micic D., Nedeljkovic I. (2019). The Amount of Weight Loss Six Months after Bariatric Surgery: It Makes a Difference. Obes. Facts.

[44] Herdy A.H., Uhlendorf D. (2011). Valores de referência para o teste cardiopulmonar para homens e mulheres sedentários e ativos. Arq. Bras. Cardiol..

[45] Ingle L., Rigby A., Brodie D., Sandercock G. (2020). Normative reference values for estimated cardiorespiratory fitness in apparently healthy British men and women. PLoS ONE.

[46] Wilms B., Ernst B., Thurnheer M., Weisser B., Schultes B. (2013). Differential Changes in Exercise Performance After Massive Weight Loss Induced by Bariatric Surgery. Obes. Surg..

